# Base editing both DNA strands in distinct editing windows with small CRISPR-associated effector Cas12f1

**DOI:** 10.1016/j.isci.2025.114033

**Published:** 2025-11-12

**Authors:** Thomas Swartjes, Evgenios Bouzetos, Belén Adiego-Pérez, Victor D. Pool, Raymond H.J. Staals, John van der Oost, Wen Y. Wu

**Affiliations:** 1 Laboratory of Microbiology, Wageningen University & Research, Wageningen, the Netherlands; 2 Department of Chemical and Pharmaceutical Biology, Groningen Research Institute of Pharmacy, University of Groningen, Groningen, the Netherlands

**Keywords:** Genetic engineering, Enzyme engineering, Biomanipulation

## Abstract

CRISPR-associated base editors have been established as genome editing tools that enable base conversions in targeted DNA sequences, without generating double-strand breaks. Here, we describe the development of new base editors based on CRISPR-Cas12f1, a miniature Cas protein of only 422 amino acids. Chimeric constructs have been generated by fusing a catalytically inactive dCas12f1, to either a cytosine deaminase or an adenine deaminase. Using these synthetic fusion proteins, systematic analyses have been performed on base editing of a target sequence on a plasmid in *Escherichia coli*. Interestingly, apart from the previously described base editing of the displaced non-target DNA strand, we also observed efficient editing of the target DNA strand. This effect was not observed for Un1Cas12f1 BEs. In addition to the small size of AsCas12f1 base editors, its unique editing profile makes it a valuable addition to the CRISPR-Cas toolbox.

## Highlights

• Miniature Cas12f1 enables design of compact base editors
• New Cas12f-based adenine and cytosine base editors were developed and tested in *E. coli*
• Cas12f base editors catalyze base conversion on both DNA strands in distinct windows
• Efficient base editing was shown on both plasmid and chromosomal DNA targets

## Graphical abstract

**Figure:**
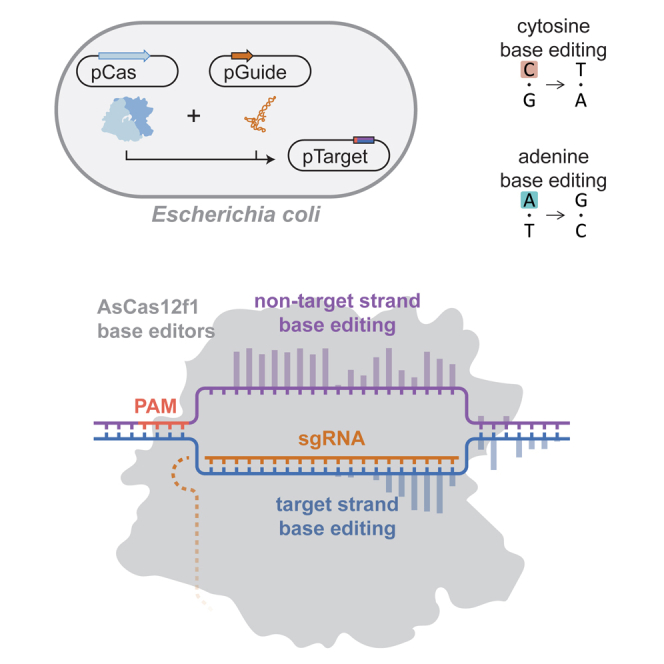

## Introduction

CRISPR-Cas is an adaptive immune system used by prokaryotic cells to defend themselves against invading genetic elements like plasmids and viruses.^1^^,^^2^ The immune system on the host chromosome typically comprises an array of clustered regularly interspaced short palindromic repeats (CRISPR) and neighboring CRISPR-associated genes encoding Cas proteins.^3^^,^^4^ Although all CRISPR-Cas systems use CRISPR-derived RNA guides (crRNA), they are divided into two distinct classes: class 1 systems with a multi-protein Cas effector complex (types I, III, and IV) and class 2 systems with a single, multi-domain effector (types II, V, and VI).^5^ The best characterized DNA-targeting CRISPR-Cas nucleases are Cas9 and Cas12a, belonging to type II and type V-A, respectively.^6^^,^^7^^,^^8^^,^^9^ After crRNA-dependent recognition of an appropriate protospacer adjacent motif (PAM),^10^ the Cas-RNA complex binds its dsDNA target through base pairing of the guide and the complementary target DNA strand, while displacing the non-target strand.^11^^,^^12^^,^^13^ After formation of a complete R-loop structure, the nuclease activity of Cas9 and Cas12a results in a double-strand break (DSB) in the target DNA.^6^^,^^8^

CRISPR-Cas systems can be heterologously expressed in a variety of hosts. The unique feature of programmable DNA target specificity by designing a matching guide has established CRISPR-Cas nucleases as highly efficient tools, with applications ranging from biotechnology (engineering of microbes and crops) to biomedicine (gene therapy and diagnostics).^14^^,^^15^ After introduction of a DSB in the target sequence, the actual outcome of the editing relies on the subsequent DNA repair mechanism, generally by non-homologous end-joining (NHEJ) or by homology directed repair (HDR).^16^ While NHEJ can disrupt a gene’s functionality,^17^ HDR allows for precise edits^16^ when a DNA repair fragment is provided. However, HDR is generally less efficient compared to NHEJ, and is usually restricted to the S and the G2 phase of the cell cycle in eukaryotic cells.^18^ In addition, the dependency of both repair systems on DSBs is considered a disadvantage, as this may result in undesired recombination events.^19^^,^^20^^,^^21^^,^^22^

Base Editing is a genome editing approach that requires neither the introduction of DSBs, nor co-delivery of DNA repair fragments.^23^ Reported base editors employ a Cas effector protein (e.g., SpCas9: 1,368 amino acids, AsCas12a: 1,307 amino acids) in which the nuclease activity has completely or partially been disrupted.^24^^,^^25^^,^^26^ This Cas effector is fused either to an adenine deaminase (e.g., TadA) or to a cytosine deaminase (e.g., APOBEC1), resulting in an adenine base editor (ABE) or a cytosine base editor (CBE), respectively. The latter usually includes an additional uracil DNA glycosidase inhibitor (UGI) to increase base editing efficiency by supressing the uracil DNA glycosylase enzyme that initiates the base excision repair pathway reversing a U:G pair back to the original C:G pair.^25^^,^^27^ After targeting and formation of an R-loop structure, base editing occurs through deamination of bases on the displaced, non-target strand, within a certain editing window. The position of base editing most likely depends mainly on the accessibility of the deaminase domain to the nucleotide(s), and hence on the overall base editor architecture.^23^ Although base editing has also been demonstrated to occur with catalytically dead effectors (dCas), substantially increased efficiencies have been obtained with base editors that use a nickase variant (nCas9).^27^^,^^28^ The nCas9 (RuvC-mutation) cleaves only the guide-complementary target strand. The nicked target strand is subsequently repaired by the mismatch repair pathway using the edited displaced strand as repair template.^23^^,^^25^^,^^27^^,^^29^ Currently existing Cas9 and Cas12a base editors are relatively large, exceeding the cargo capacity of viral vector delivery systems such as adeno assocciated virus (AAV), thereby obstructing their use for *in vivo* therapeutic editing.^30^ One way to overcome this limitation would be to use smaller effector proteins for base editing.

One recently characterized small Cas protein is Cas12f1^31^^,^^32^ that functions as an asymmetrical homodimer. ^33,34^ The Cas12f1 dimer, guided by a single crRNA-tracrRNA pair, recognizes a 5′ T-rich PAM, forms an R-loop and cleaves its dsDNA target. Cleavage of dsDNA occurs through a single RuvC domain of the Cas12f1.1 protomer.^33^^,^^34^ Cas12f1 homologs range from 400 to 700 residues, and they have been used successfully for genome editing, including base editing, in mammalian and plant cells, especially after optimization by protein and sgRNA engineering.^35^^,^^36^^,^^37^^,^^38^^,^^39^^,^^40^^,^^41^^,^^42^

Here we describe base editors (CBEs and ABEs) using the dCas12f protein of *Sulfoacidibacillus thermotolerans* (previously called *Acidibacillus sulfuroxidans*). At present, Cas12f1 base editors are the most compact base editors within the CRISPR-Cas toolbox, with a length of 2.3 Kbp and 1.9 Kbp for CBE and ABE, respectively. As they easily fit into an AAV capsid, they would allow for *in vivo* gene therapy.^30^ The here developed Cas12f1 BEs were characterized in *E. coli,* resulting in high base editing efficiencies with a broad base editing window of the non-target strand. Interestingly, we describe the, efficient editing of bases on the target strand as well, which was only recently described.^43^.

## Results and discussion

### AsCas12f1 target strand base editing on plasmid DNA

**Figure 1:**
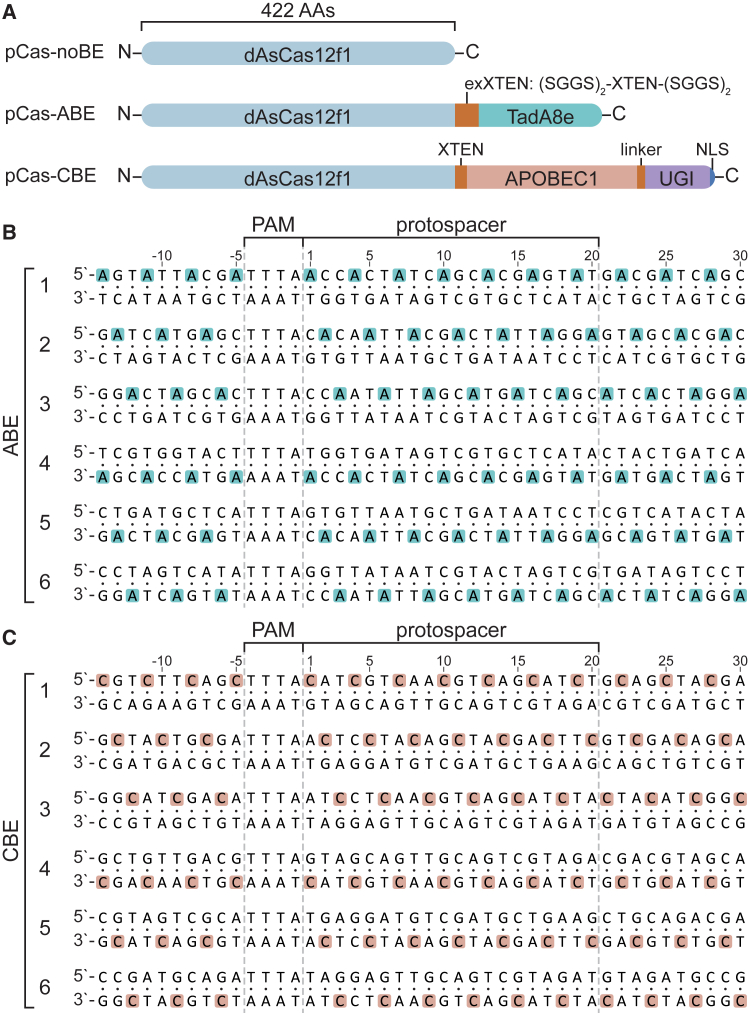
Protein fusion layout and nucleotide tiling strategy (A) Schematic overview of the fusion protein encoded on each pCas plasmid. The width of each element is proportional to their amino acid sequence length. C and N termini are indicated for each fusion protein. Linkers are indicated in orange. AAs, amino acids; NLS, nuclear localization signal. (B and C) Target sequences of ABE (B) and CBE (C) pTarget plasmids. The tiled bases are shown in blue (ABE) or pink (CBE).

To establish base editing with the AsCas12f1 protein, we used a set of three plasmids: pCas, pTarget, and pGuide. The pCas plasmids encode a catalytically inactivated variant (D225A) of AsCas12f1,^44^ fused to either adenine deaminase TadA8e or cytosine deaminase APOBEC1 and UGI for adenine and cytosine base editing, respectively (Figure 1A). The pTarget plasmids each contain a 10bp-PAM-protospacer-10bp region with a target (A or C) nucleotide in every third position, except in the PAM (Figures 1B and 1C, and Table S1).

For each protospacer, a pGuide plasmid was used expressing the corresponding single guide RNA (sgRNA).^44^ *Escherichia coli* cells harboring pTarget and pGuide were transformed with the pCas plasmid, after which they were incubated to allow base editing. Cells were sampled after 24, 48, and 72 h, followed by target sequence amplification, and subsequent analysis by deep sequencing. We scored base editing at all positions, inferring either non-target (NTS) or target strand (TS) editing depending on the observed base conversion (Figure S1).

**Figure 2:**
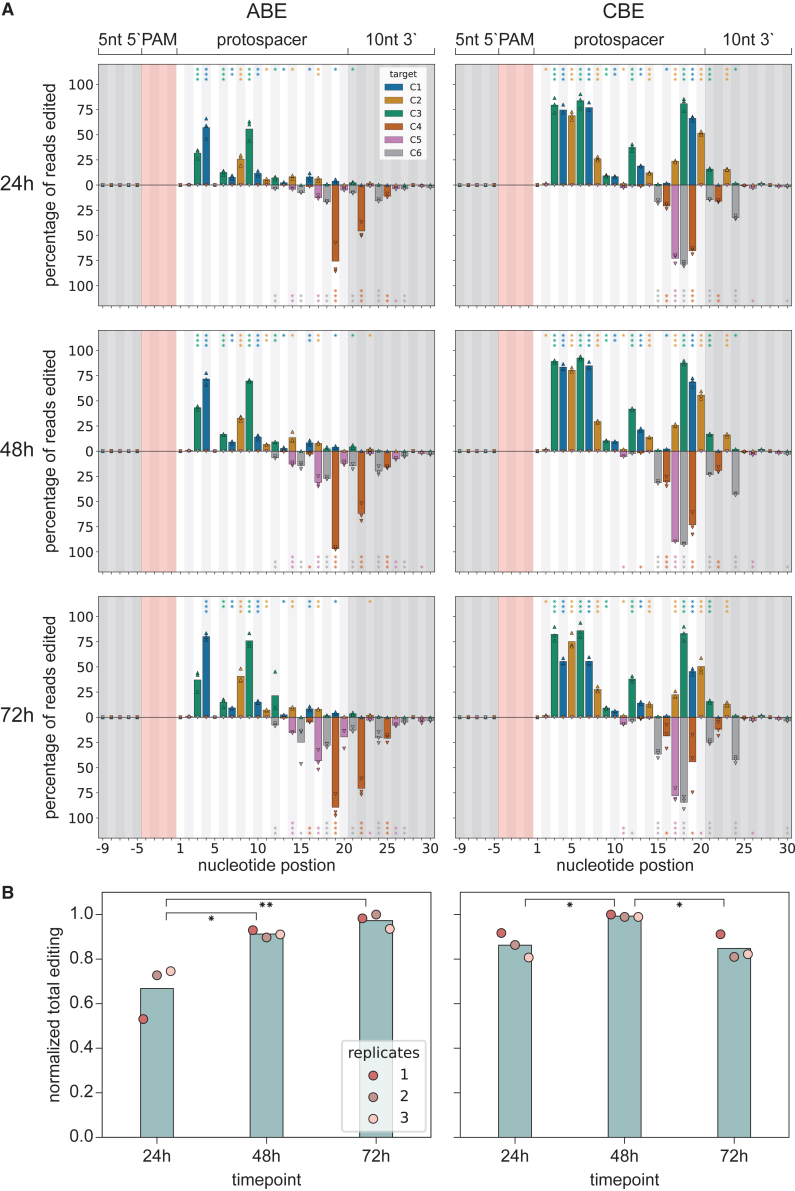
ABE and CBE editing at different positions and durations (A) Bar charts showing base editing activity at tiled positions (Figure 1), combining data from all protospacers for ABE and CBE at three time points (plots per pTarget in Figure S1). The data from each biological replicate are shown as colored triangles (pointed up: NTS, pointed down: TS), while the bars indicate the average of the three replicates. The pink-shaded area indicates the PAM, and the gray-shaded areas are upstream from the PAM and downstream from the 20 nt we refer to as protospacer. Eight-spoked asterisks (✳) indicate beta-binomial adjusted *p* values (Benjamini-Hochberg false discovery rate correction) compared to non-targeting controls. ✳*p* < 0.05, ✳✳*p* < 0.01, and ✳✳✳*p* < 0.001. (B) Bar charts of the total editing (over all positions) by time point. The dots show total editing for single replicates, with the bars indicating the average across the three biological replicates. Eight-spoked asterisks (✳) indicate adjusted *p* values (Tukey’s range *post hoc* test) compared between time points. ✳*p* < 0.05 and ✳✳*p* < 0.01.

We found highly efficient base conversion at many positions throughout the target sequences, for both adenine and cytosine base editing (Figure S1). We detected neglectable base conversion numbers in conditions without a deaminase or with a non-targeting sgRNA (Figure S2A). When considering the combined data from the different pTarget plasmids, the CBE specifically caused efficient base conversion at positions throughout the NTS of the protospacer (Figure 2A). Base conversion by the ABE was mostly restricted to the PAM proximal half for the NTS. Interestingly, we also observed efficient base conversion in the PAM-distal half of the protospacer on the TS for both base editors (Figure 2A). Moreover, target strand (TS) editing was observed concurrently with non-target strand (NTS) editing (Extended Supplementary Files). After 48 h, the most frequent mutation for the CBE was C10T–G17A at 77.6% ± 0.4% (target C6), while the most common ABE-induced mutations were A8G, T19C, and T22C (target A4), collectively occurring at 37.4% ± 4.4% (Tables S1 and S2).

These findings deviate substantially from those reported for wildtype ABEs that use Cas12f1 from *Candidatus Woesearchaeota* or an uncultured archaeon, where optimal editing is only reported for NTS positions in the PAM proximal part of the protospacer.^37^^,^^45^ This discrepancy may be due to structural differences between the Cas12f1 proteins or the various TadA mutants used in different studies.

Base editing studies have commonly shown non-target strand base editing and therefore it was thought that only NTS nucleotides are available as deamination substrate due to TS-sgRNA heteroduplex formation.^23^^,^^24^^,^^29^ In most base editing applications, a nickase version of the Cas9 nuclease is used to cleave only the TS (e.g., SpCas9 D10A). In that way, retention of the edited NTS sequence is favored as template strand during DNA repair, probably concealing base editing of the TS.^24^ Untill recently, base editing of the TS had only been reported using SpCas9 (D10A) cytosine base editing and was most prevalent (up to 6.3% editing) outside of the protospacer region or at off-target sequences.^46^ Reported TS cytosine base editing at on-target sites was less than 1% at individual nucleotides.^46^ A recent article however also describes TS base editing using Un1Cas12f1 engineered for higher activity.^43^ Apart from efficient base editing of the TS, we also detected substantial editing outside of the protospacer in the PAM-distal end for both ABE and CBE (Figure 2A). Similarly to TS base editing, such conversions downstream of the target sequence have rarely been reported.^43^^,^^46^ This may suggest that the melting of the dsDNA may expand beyond the sgRNA/target-strand heteroduplex, likely mediated by the AsCas12f1 protein, as that would expose downstream nucleotides to the deaminase.

Our sequence analyses indicate that base editing is highly sensitive to the neighboring nucleotide sequence. For instance, C-to-T editing by CBE on pTarget-5 resulted in very high editing at position 10 of the NTS, while editing at the same position in pTarget-1 is far lower (Figure S1). The results presented here therefore show where editing can happen, but do not exclude that editing might also occur elsewhere in a different sequence context.

When comparing total base editing levels over time, we found that ABE catalyzed A-to-G conversion increased through time (Figure 2B). However, in the case of CBE, C-to-T conversion increases from 24 to 48 h but then drops from 48 to 72 h (Figure 2B). We attribute this to the inherit cell toxicity of the APOBEC1 cytidine deaminase.^47^^,^^48^^,^^49^ Cells that disrupt APOBEC1 expression through random mutations are thought to have a substantial fitness advantage. After 72 h, these cells (for which we expect lower cytosine base editing levels) have therefore been enriched, skewing the editing results.

### AsCas12f1 base editing on genomic targets

**Figure 3:**
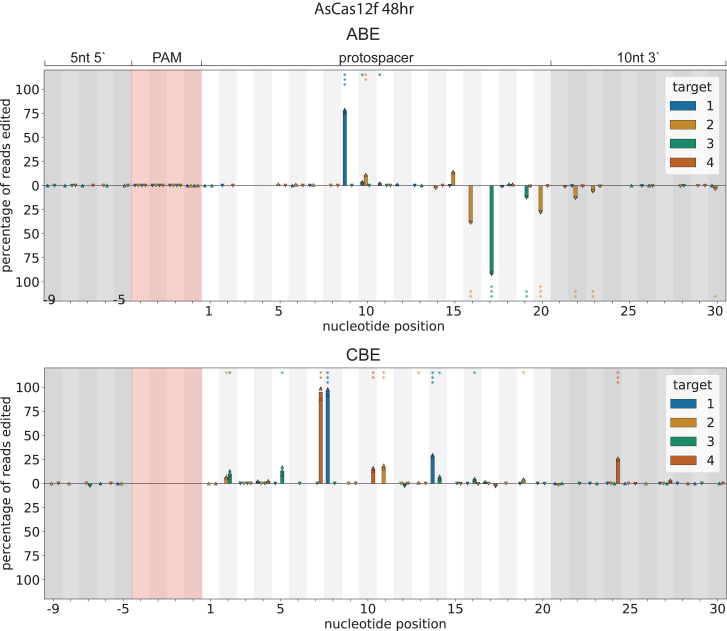
ABE and CBE editing on the *E. coli* genome Bar charts showing base editing activity on genomic targets, combining data from four targets, 1 to 4 (Tables S3 and S5). The data from each biological replicate are shown as a triangle, while the bars indicate the average of the three replicates. Triangles pointing up or down refer to NT and T strand editing, respectively. The pink-shaded area indicates the PAM, and the gray-shaded areas are upstream from the PAM and downstream of the protospacer (20 nt). Above the *x* axis refers to NT strand editing and below the *x* axis refers to T strand editing. *p* values are indicated with ✳*p* < 0.05, ✳✳*p* < 0.01, and ✳✳✳*p* < 0.001.

After demonstrating TS base editing using both AsCas12f1 ABE and CBE on plasmid targets, we next evaluated their activity on genomic targets in *E. coli*. Four non-essential genomic loci were selected based on the presence of a suitable target nucleotide substrate within the estimated base editing window (see Table S5 for spacer sequences). Editing efficiency was assessed at 24 h (Figure S3) and 48 h after introduction of the BE plasmids (Figure 3). Compared to plasmid targets, both ABE and CBE showed a narrower base editing window on genomic targets. However, the observed edits fell within the previously defined editing window (Figure 2A). An exception was observed for CBE, where a C-to-T conversion at position 24 on the NTS was detected, which was not previously observed for plasmid editing. Interestingly, ABE base edited both the TS and NTS on genomic loci, whereas CBE edits were restricted to the NTS. These findings point to several context-dependent factors that may influence base editing outcomes in genomic DNA. First, the reduced editing window on chromosomal targets may reflect differences in DNA topology, DNA accessibility, or replication dynamics compared to episomal plasmids. Second, the observed strand bias, particularly the absence of TS editing for CBE, suggests potential differences in deaminase accessibility.

### Un1Cas12f1 ABE and CBE

**Figure 4:**
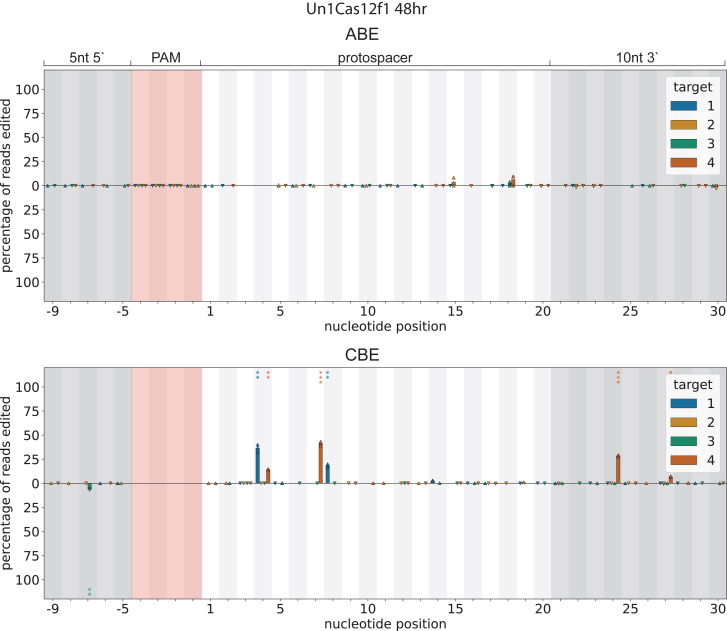
Un1Cas12f1 ABE and CBE editing *E. coli* genome Bar charts showing base editing activity on genomic targets, combining data from four targets, 1 to 4 (Table S5). The data from each biological replicate are shown as a triangle, while the bars indicate the average of the three replicates. Triangles pointing up or down refer to NT and T strand editing. The pink-shaded area indicates the PAM, and the gray-shaded areas are upstream from the PAM and downstream of the protospacer (20 nt). Above the *x* axis refers to NT strand editing, and below the *x* axis refers to T strand editing. *p* values are indicated with ✳*p* < 0.05, ✳✳*p* < 0.01, and ✳✳✳*p* < 0.001.

To investigate whether TS base editing is a unique feature of AsCas12f1 base editors or a property more broadly shared among dimeric Cas12f1 proteins, we constructed ABE and CBE versions of Un1Cas12f1 using the same deaminases relying on linker and fusion architecture as in the AsCas12f1 constructs. These BEs were tested on the same four *E. coli* genomic targets used in the AsCas12f experiments (Figure 4). Base editing was observed only with the Un1Cas12f1 CBE. C-to-T conversions were detected on the NTS at positions 4, 7, 8, and 24, although with overall lower efficiency compared to AsCas12f1 CBE. While edits at positions 7, 8, and 24 were shared between both AsCas12f1 and Un1Cas12f1 CBEs, position 4 editing was unique to Un1Cas12f1 CBE. No base editing activity was observed with the Un1Cas12f1 ABE at any of the tested sites.

These differences in editing efficiency and positional outcomes likely reflect intrinsic differences in the protein structure between the AsCas12f1 and Un1Cas12f1 variants. Previous studies of Un1Cas12f ABE in mammalian cells have reported efficient A-to-G editing on position 3 and 4. However, these base editors used a different version of the TadA and fused it to the N terminus of Cas12f.^37^^,^^45^ Similarly, an N-terminal fusion Un1Cas12f CBE showed a broad editing window and editing efficiency of up to 50% in mammalian cells.^50^ While we have not observed TS editing for Un1Cas12f BEs in the current study, a recent publication does report TS editing with a CBE using an engineered version of Un1Cas12f1.^43^ This suggests that the details of protein and protein fusion design affect editing efficiency and DNA strand preference.

**Figure 5:**
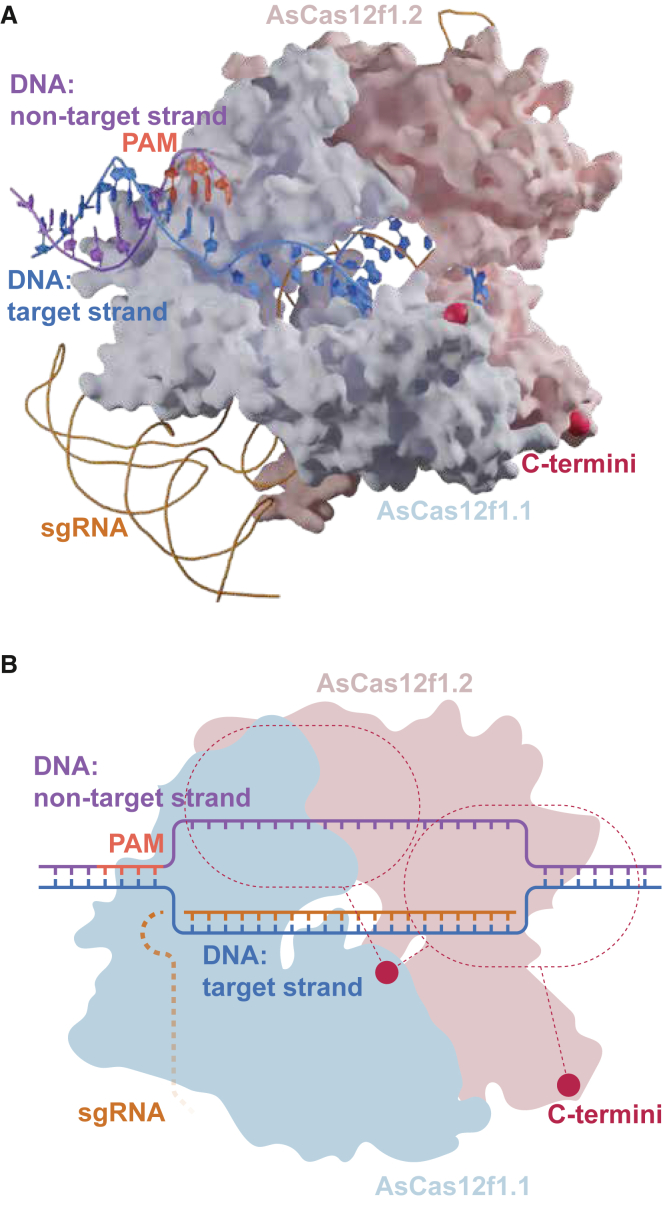
Protein structure of AsCas12f1 and working model of AsCas12f1 base editors (A) AsCas12f1 protein structure (PDB 8J12). C termini are indicated with red dots to indicate where the deaminase domains were fused. (B) Working model for AsCas12f1 base editing where the dashed red lines indicate which C terminus we think the deaminase is fused to that is responsible for edits within the pill-shaped areas.

In conclusion, we have constructed compact ABE and CBE using a catalytically inactive AsCas12f1. These BEs are half the size of those relying on Cas9, offering advantages for delivery in size-constrained vectors such as AAV. When tested in *E.* *coli*, we observed high levels of base editing when targeting plasmid vectors, both on the NTS as well as on the TS (Figure 2A) of the DNA sequence. Furthermore, base editing was observed across an extended window that includes positions downstream of the protospacer on both DNA strands. What remains unclear is whether NTS and TS base editing is carried out by a single deaminase or by two seperate deaminases, each fused to a Cas12f monomer (Figure 5). The observed broad editing range increases flexibility of using BEs in therapeutically relevant applications, particularly in scenarios constrained by PAM availability or narrow editing windows. Specific examples include disrupting splice sites, modifying transcription factor binding sites, or targeting multiple residues within protein–protein interaction domains. Moreover, we compared AsCas12f1 ABE and CBE with Un1Cas12f ABE and CBE targeting four genomic loci *in E. coli.* Notably, target strand base editing was observed only for AsCas12f ABE and AsCas12f1-based BEs exhibited higher overall editing efficiency. As such, this study provides a descriptive characterization of compact Cas12f base editors, offering new insight into their on-target editing capabilities and underscoring the importance of evaluating potential off-target activity on both DNA strands. Together, our findings highlight the wide range of possible base editing outcomes and further establish Cas12f1-based editors as a valuable addition to the genome editing toolbox. Overall, our results show that alternative Cas effectors and derived synthetic variants may have unexpected and unique features that are not only interesting for fundamental reasons, but that might also be useful for certain applications.

### Limitations of the study

The base editing activities described in this work were characterized exclusively in *E. coli*. While this bacterial system allowed for systematic evaluation of Cas12f BEs, it does not fully replicate the chromatin context, DNA repair pathways, or cellular environment of eukaryotic cells. Future studies will be required to evaluate these editors in eukaryotic cells, particularly in therapeutically relevant contexts.

Due to the context dependency of base editing, the base editing windows reported here are likely specific to the sequences that were tested. Therefore, the current results show where editing can take place but do not conclusively exclude the possibility of editing occurring in different positions for different target and guide sequences.

## Resource availability

### Lead contact

Further information and requests for resources and reagents should be directed to the lead contact, Wen Y. Wu (w.y.wu@rug.nl).

### Materials availability

Important plasmids have been deposited at Addgene and are publicly available as of the date of publication. Accession numbers are listed in the key resources table.

### Data and code availability

• The deep-sequencing data have been deposited at NCBI and are publicly available as of the date of publication. The accession number is listed in the key resources table.
• Custom scripts and analyzed data including outcomes of statistical testing have been deposited at Mendeley Data and are publicly available as of the date of publication. The link to the Mendeley Data entry is listed in the key resources table.
• No additional resources are reported.

## Acknowledgments

This research was supported by research grants of the 10.13039/100012838Dutch Research Council (10.13039/501100003246NWO; BBOL-737.016.005, Spinoza SPI 93–537, and Gravitation 024.003.019), as well as of the 10.13039/100010663European Research Council (10.13039/100008698ERC-AdG-834279).

## Author contributions

T.S., E.B., R.H.J.S., J.v.d.O., and W.Y.W. conceived and designed the study. T.S., E.B., B.A.P., V.D.P., and W.Y.W. conducted all the experimental work and analyzed the data. T.S. performed the NGS sequencing experiments and analysis. T.S., E.B., J.v.d.O., and W.Y.W. wrote the manuscript with input from all authors.

## Declaration of interests

R.H.J.S. and J.v.d.O. are shareholders and members of the scientific advisory board of Scope Biosciences B.V. J.v.d.O. is a scientific advisor of NTrans Technologies and Hudson River Biotechnology. T.S., R.H.J.S., J.v.d.O., and W.Y.W. are inventors on CRISPR-Cas-related patents and patent applications.

## STAR★Methods

### Key resources table

**Table:** 

REAGENT or RESOURCE	SOURCE	IDENTIFIER
**Bacterial and virus strains**		

DH5-alpha chemical competent cells	This study	N/A
BW25113 Δcas3-PcasA::PJ23119 ΔPlacI-lacZ:cat	Chase Beisel Lab	N/A

**Critical commercial assays**		

Q5 Hifi 2X Mastermix	NEB	M0492
Hifi DNA Assembly Master Mix	NEB	E2621S
QIAxcel DNA high-sensitivity kit	Qiagen	929012
DNA Clean & Concentrator-5	Zymo Research	D4004
Qubit™ dsDNA, BR	Thermo Fischer	Q33266
Qubit™ dsDNA, HS	Thermo Fischer	Q33230
AMPure XP Beads	Beckman Coulter	A63881
NEBNext® Ultra™ II DNA Library Prep Kit for Illumina®	NEB	E7645

**Deposited data**		

Raw data	this study	Bioproject: PRJNA1109600, NCBI
Analyzed data	this study	Available from Mendeley Data: https://doi.org/10.17632/f92c7ysmys.1

**Oligonucleotides**		

Oligonucleotides	IDT	Table S4

**Recombinant DNA**		

Plasmid Vectors	This study	List of plasmids in Table S3. Plasmid sequences listed on Mendeley Data: https://doi.org/10.17632/f92c7ysmys.1

**Software and algorithms**		

Python 3.13.2	N/A	N/A
Numpy 2.2.5	N/A	N/A
Pandas 2.2.3	N/A	N/A
Seqprep 1.3.2–7	St. John^51^	N/A
Fastp 0.20.1	Chen et al.^52^	N/A
SciPy 1.15.3	Virtanen et al.^53^	N/A
Statsmodels 0.14.4	Seabold and Perktold^54^	N/A
Matplotlib 3.10.0	N/A	N/A
Seaborn 0.13.2	N/A	N/A
Custom scripts	this study	Available from Mendeley Data: https://doi.org/10.17632/f92c7ysmys.1

**Other**		

MASTERBLOCK®, 96 WELL, 2 ML, PP, V-BOTTOM	Greiner BIO-ONE	780271
Gas-permeable membrane	Diversified Biotech	BERM-2000
M9TG media, [1xM9 salts (Sigma), 10 g/L tryptone (Oxoid), 5 g/L glycerol (Acros)]	This study	N/A

### Experimental model and study participant details

#### Bacterial strains and growth conditions

*Escherichia coli* strains used in the current study.

• **DH5α**: F^−^ endA1 glnV44 thi-1 recA1 relA1 gyrA96 deoR nupG purB20 φ80d[lacZΔM15] Δ(lacZYA-argF)U169, hsdR17(r_K_^-^m_K_^+^) λ^-^
• **DH10B**: F- endA1 recA1 galE15 galK16 nupG rpsL ΔlacX74 Φ80lacZΔM15 araD139 Δ(ara,leu)7697 mcrA Δ(mrr-hsdRMS-mcrBC) λ-
• **BW25113 ΔPlacI-lacZ::cat**: Δ(*araD-araB*)*567* Δ(*rhaD-rhaB*)*568 ΔlacZ4787* (:rrnB-3) *hsdR514 rph-1* ΔPlacI-lacZ:cat

These strains were not authenticated.

*E. coli* strains were routinely cultured at 37°C and 220 rpm, unless otherwise specified, in either lysogeny broth (LB) [10 g/L peptone (Oxoid), 5 g/L yeast extract (BD), 10 g/L NaCl (Acros)] or M9TG minimal medium [1xM9 salts (Sigma), 10 g/L tryptone (Oxoid), 5 g/L glycerol (Acros)]. Plasmids were maintained with ampicillin (100 μg/mL), chloramphenicol (35 μg/mL), and/or kanamycin (50 μg/mL) as needed.

### Method details

#### Bacterial strains and growth conditions

Bacterial strains used for the cloning and propagation of plasmids in the current study are *E. coli* DH5α and DH10β. To assess base editing, we used *E. coli* strain BW25113 lacking the *lacI*, *lacZ* genes and the type I-E CRISPR-Cas system.^55^ The *E. coli* strains were routinely cultured at 37°C and 220 rpm, unless otherwise specified, in either lysogeny broth (LB) [10 g/L peptone (Oxoid), 5 g/L yeast extract (BD), 10 g/L NaCl (Acros)] or M9TG minimal medium [1xM9 salts (Sigma), 10 g/L tryptone (Oxoid), 5 g/L glycerol (Acros)]. Plasmids were maintained with ampicillin (100 μg/mL), chloramphenicol (35 μg/mL), and/or kanamycin (50 μg/mL) as needed.

#### Plasmid construction

All plasmids and oligo nucleotides used in this study can be found in Tables S3 and S4, respectively. For this study, we used a three-plasmid system (pCas, pGuide and pTarget) as described in previous studies.^55^ The *asdcas12f1* gene, with a mutation that results in a catalytically inactive nuclease (D225A),^44^ was amplified from a synthetically synthesized AsCas12f1 CRISPR locus (IDT gBlock, Table S3) and inserted into the pBAD33^56^ vector backbone under the control of the constitutive J23108 promoter to construct pCas-noBE. The pCas base editor plasmids (pCas- ABE/CBE) were constructed from this negative control plasmid by fusing TadA8e^57^ (ABE) or APOBEC1 (CBEs) to the C-terminal end of AsdCas12f1 (Figure 1A). For CBEs, a uracil glycosylase inhibitor (UGI) was fused to the C-terminal end of APOBEC1. When cloning pCas plasmids, especially pCas-CBE, we observed many mutations arising in the coding sequences of the BEs. This suggested that the cells experienced toxicity by the high expression levels of these proteins. To decrease toxicity and the resulting evolutionary pressure, we fused the last three amino acids (LVA) of the LVA ssrA degradation tag^58^ to the C-terminus of all AsdCas12f1 (fusion) proteins, encoded by in the pCas plasmids. Un1Cas12f1 pCas vectors were assembled by replacing AsCas12f1 with Un1Cas12f1 CRISPR locus (Table S3). A pGuide-RFP-entry plasmid was generated by inserting a sgRNA^44^ downstream of the constitutive J23119 promoter in a pBAD18 backbone.^56^ Spacer sequences were assemblexd into linear dsDNA by annealing ssDNA oligonucleotides and were inserted to the pGuide-RFP-entry plasmid by BbsI restriction and ligation. All vectors were sequence-verified by Sanger’s sequencing and or Nanopore sequencing.

Notably, when pGuide-RFP-entry was re-cultivated from glycerol stock, it presented genetic instability which translated to insertion of IS element in the sgRNA-scaffold, or deletion of short nucleotide stretches in sgRNA-scaffold and loss of RFP color.

To generate pUn1Guide-RFP we replaced the AsCas12f1 sgRNA locus with an Un1Cas12f1 sgRNA locus (Table S1). The pTarget Entry contains a *gfp* and a *mrfp* gene under the P_lacIQ_ and P_TAQ_ promoters, respectively, in a pAU66 plasmid backbone.^59^ Target sequences were similarly assembled as linear dsDNA by annealing ssDNA oligonucleotides and inserted in place of the *mrfp* gene by AatII/KpnI restriction ligation.

#### Base editing assay

*E. coli* cells (adapted BW25113, as described above) harboring pGuide plasmids and their corresponding pTarget plasmids were made chemically competent and transformed with the different pCas plasmids. To this end, 45 μL of competent cells where mixed with 2 μL 50 ng/μL of pCas plasmid DNA, kept on ice for 30min, heat-shocked at 42°C for 30s, kept on ice for 2min. Then, 150 μL LB without antibiotics was added and the cell suspension was transferred to a 96-wells 2 mL deep-well plate (Greiner) and incubated for 1h at 37^0^C with short-stroke shaking at 900rpm for recovery. Different biological replicates originate from distinct batches of competent cells and were transformed with pCas separately. After recovery, the transformation mix was diluted: 2 μL + 198 μL in M9TG medium in 96-wells 2 mL deep-well plate. The deep-well plate was then sealed using a gas-permeable membrane (Diversified Biotech, Breathe-EASIER) and grown incubated at 37°C with short-stroke shaking at 900rpm for 24h. For subsequent time points (48h and 72h), the cells were serially diluted twice, mixing 2 μL with 198 μL sterile PBS 1X buffer (ThermoFischer, Cat. No. 10010023) in a deep-well plate. The bacteria were then incubated to grow 24h at 37°C. For the 72h timepoint, the previous step was done twice. For each timepoint, the remainder of the undiluted bacterial cultures was stored at −20°C. To assess base edition on genomic loci, 15 μL of chemically competent *E. coli* cells harboring only the corresponding pGuide plasmid were transformed with 1.5 μL 20 ng/μl pCas plasmids. Following transformation, *E. coli* cells recovered and were cultured as described above.

#### NGS sample preparation

Diluted by mixing 10 μL of the stored samples with 90 μL mQ water and again stored at −20°C for one week. At this point, due to a building-wide power outage, the stored diluted samples thawed and remained at ∼22°C for ∼1.5 weeks, before analysis. We verified through Sanger sequencing comparable base editing before and after the power outage incident. A 150bp region surrounding the protospacer was PCR amplified by using 2 μL diluted sample in 25 μL PCR reactions using Q5 High-Fidelity 2X Master Mix (NEB). Forward primers used in the PCR reactions were internally barcoded using six additional nucleotides to distinguish between samples. PCR products with verified DNA concentrations were quantified by capillary gel electrophoresis using the Qiagen QIAxcel DNA high-sensitivity kit. PCR products were pooled in groups of 12, adjusting volumes to obtain equal concentrations between pooled conditions. Pooled DNA was purified using the Zymo kit DNA Clean & Concentrator-5 (D4004) according to manufacturer’s protocol, eluting in 15 μL mQ water. DNA concentrations were measured using Qubit dsDNA BR (Thermo Fischer Scientific), and DNA was further pooled in groups of 5 DNA pools, adjusting volumes to obtain equal concentrations between pooled conditions. This resulted in one pool for each replicate for which we verified roughly equal concentrations and purity by agarose gel electrophoresis. We then sent these three pools to Eurofins Genomics as three individual samples for adapter ligation, and subsequent NovaSeq 6000 paired-end Illumina sequencing.

For base editing targeting the genome of *E. coli*, ∼100bp region surrounding the protospacer sequence was PCR-amplified using 2 μL of sample in 50 μL PCR reactions using Q5 High-Fidelity 2X Master Mix (NEB). PCR products were analyzed by gel electrophoresis to confirm expected amplicon sizes and purified with AMPure XP beads (Beckman Coulter) at a 1:1.8 (sample:beads) ratio. Purified products were quantified using the Qubit dsDNA HS Assay Kit (Thermo Fisher Scientific) on a BioTek Synergy H1 plate reader (excitation at 485 nm, emission at 520 nm). A total of 50 ng of the pooled sample was prepared for next-generation sequencing (NGS) using the NEBNext Ultra II DNA Library Prep Kit for Illumina (NEB) with xGen Stubby Adapters and UDI Primer Pairs (IDT). Library preparation was performed according to the manufacturer’s protocol, with the USER enzyme cleavage step omitted. A 1:1.8 (sample:beads) AMPure XP bead cleanup was used at every purification step. Final libraries were verified and quantified using a 4200 TapeStation system with D1000 ScreenTape (Agilent). Libraries were sequenced on an Illumina NextSeq 2000 using a 77 bp single read protocol at the European Research Institute for the Biology of Aging, University Medical Center Groningen.

#### NGS data analysis

To analyze the deep-sequencing data of plasmid editing, paired-end reads were programmatically merged using seqprep.^51^ Then, the reads belonging to the different conditions were organized in separate files based on their internal barcodes. Reads were filtered out if they did not match the expected pattern of starting with a forward barcode followed by a forward primer annealing part and ending with the reverse primer annealing part. Reads with low confidence base-calls (error probability of 0.01 or greater) were removed as well. We then checked whether reads matched other pTarget plasmids (allowing for base editing mutations) instead of the pTarget associated with their barcode, and if so, discarded the reads. Finally, we analyzed the resulting reads for base editing at each position. The resulting base editing efficiencies for each target plasmid were quantified and ranked (see Data S1). The scripts used were created in-house and will be included in the online version of the article.

Chromosomal editing single-read Illumina data was initially quality filtered using fastp (qualified_quality_phred: 20, --unqualified_percent_limit: 30, length_required: 50). Reads were then dereplicated, and reads with different barcodes were organized in separate files. The reads were then compared to the original target sequences to score base editing. These editing scores were then used to analyze and visualize the results.

### Quantification and statistical analysis

#### Statistical testing

The number of biological replicates for each condition is listed together with the outcomes of statistical testing and analyzed data on Mendeley data (link in key resources table). For all plasmid editing and AsCas12f1-based editing of chromosomal targets, three biological replicates were used. In some conditions where UnCas12f1 was used to edit chromosomal targets, one culture failed to grow resulting in two remaining biological replicates. Biological replicates arise from separate colonies upon transformation of pGuide and (in case of plasmid editing pTarget) plasmids and encompass the sequencing data from the resulting bacterial cultures. Figure captions list the definition of center and dispersion.

For each base position, editing rates were modeled using beta-binomial distributions to account for overdispersion. Likelihood ratio tests compared a null model assuming equal editing distributions between treatment and non-targeting control conditions against an alternative model with condition-specific parameters. *p*-values were adjusted using Benjamini-Hochberg false discovery rate correction for multiple testing across all positions.

To compare cumulative editing at the different timepoints, Shapiro-Wilk test and Levene’s test were used to assess normality and equality of variances. One-way ANOVA was then used and followed up by a Tukey’s range post-hoc test for pairwise comparisons. The scripts used for the statistical analyses as well as a list of the statistical outcomes are included with the online manuscript.

## Supplemental information

Document S1. Figures S1–S4 and Tables S1–S5

Data S1. Summary table of base editing efficiency on plasmid target DNA
