# Supplementary material for: Base editing both DNA strands in distinct editing windows with small CRISPR-associated effector Cas12f1
Source: iScience. 2025 Nov 12;28(12):114033. doi: 10.1016/j.isci.2025.114033 (PMC12702181; doi:10.1016/j.isci.2025.114033)
Supplement: Document S1. Figures S1–S4 and Tables S1–S5 [file mmc1.pdf]

iScience, Volume *28*

Supplemental information

Base editing both DNA strands in distinct editing windows with small CRISPR-associated effector Cas12f1

Thomas Swartjes, Evgenios Bouzetos, Belén Adiego-Pérez, Victor D. Pool, Raymond H.J. Staals, John van der Oost, and Wen Y. Wu

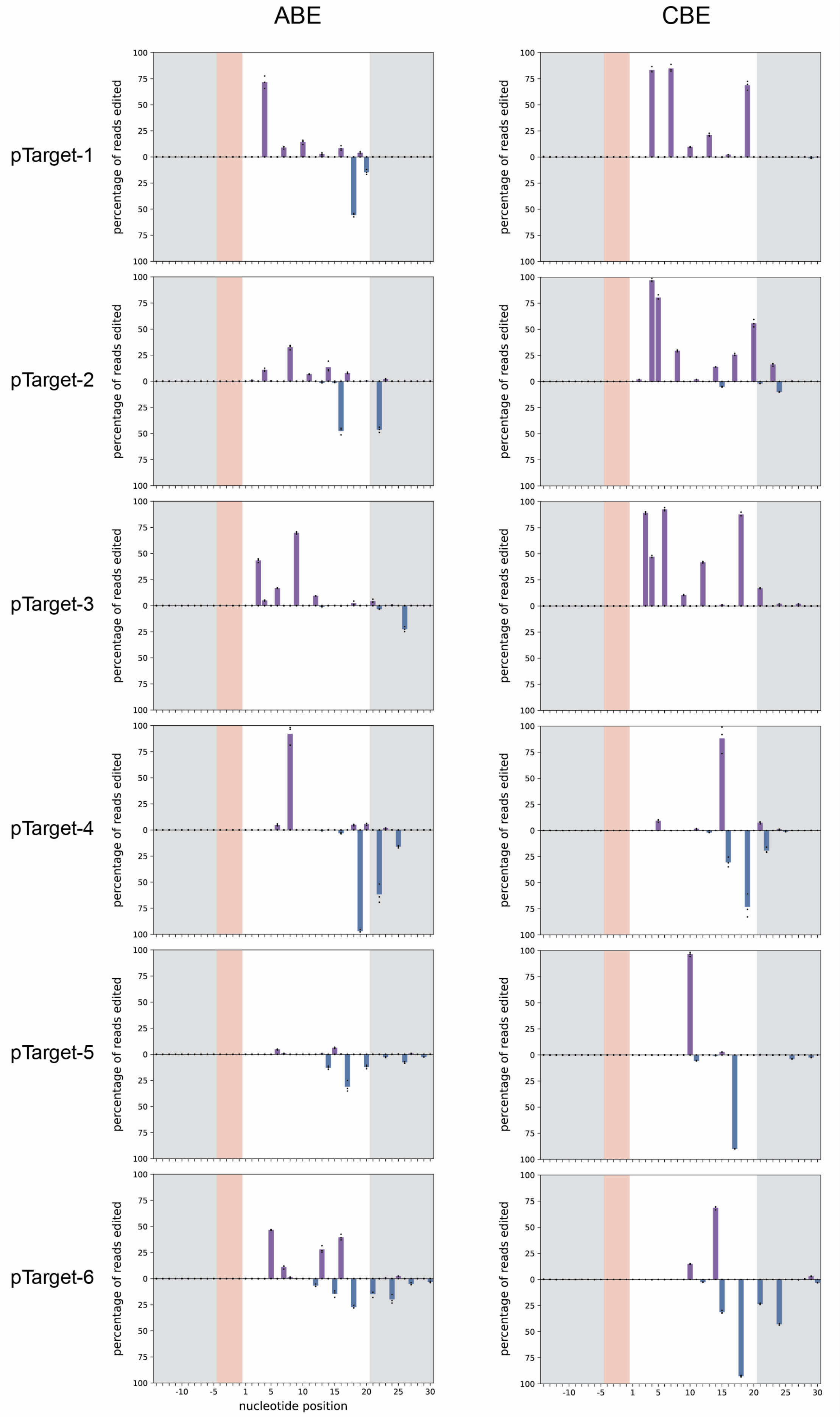
ABE
CBE
pTarget-1
pTarget-2
pTarget-3
pTarget-4
pTarget-5
pTarget-6
percentage of reads edited
nucleotide position
100
75
50
25
0
-10
-5
1
5
10
15
20
25
30

Supplementary Figure 1. Base editing at individual pTarget plasmids. Bar-charts showing base editing activity at positions in single pTarget plasmids for ABE and CBE after 48h. The data from each biological replicate is shown as black dots, while the bars indicate the average of the three replicates. The pink-shaded area indicates the PAM, the grey-shaded areas are upstream from the PAM and downstream from the 20nt we refer to as protospacer. Purple bars: non-target strand editing. Blue bars: target strand editing. In contrast to Figure 2A, not all positions could theoretically be edited because only a subset contains an adenine or cytosine that could be converted through ABE or CBE respectively.

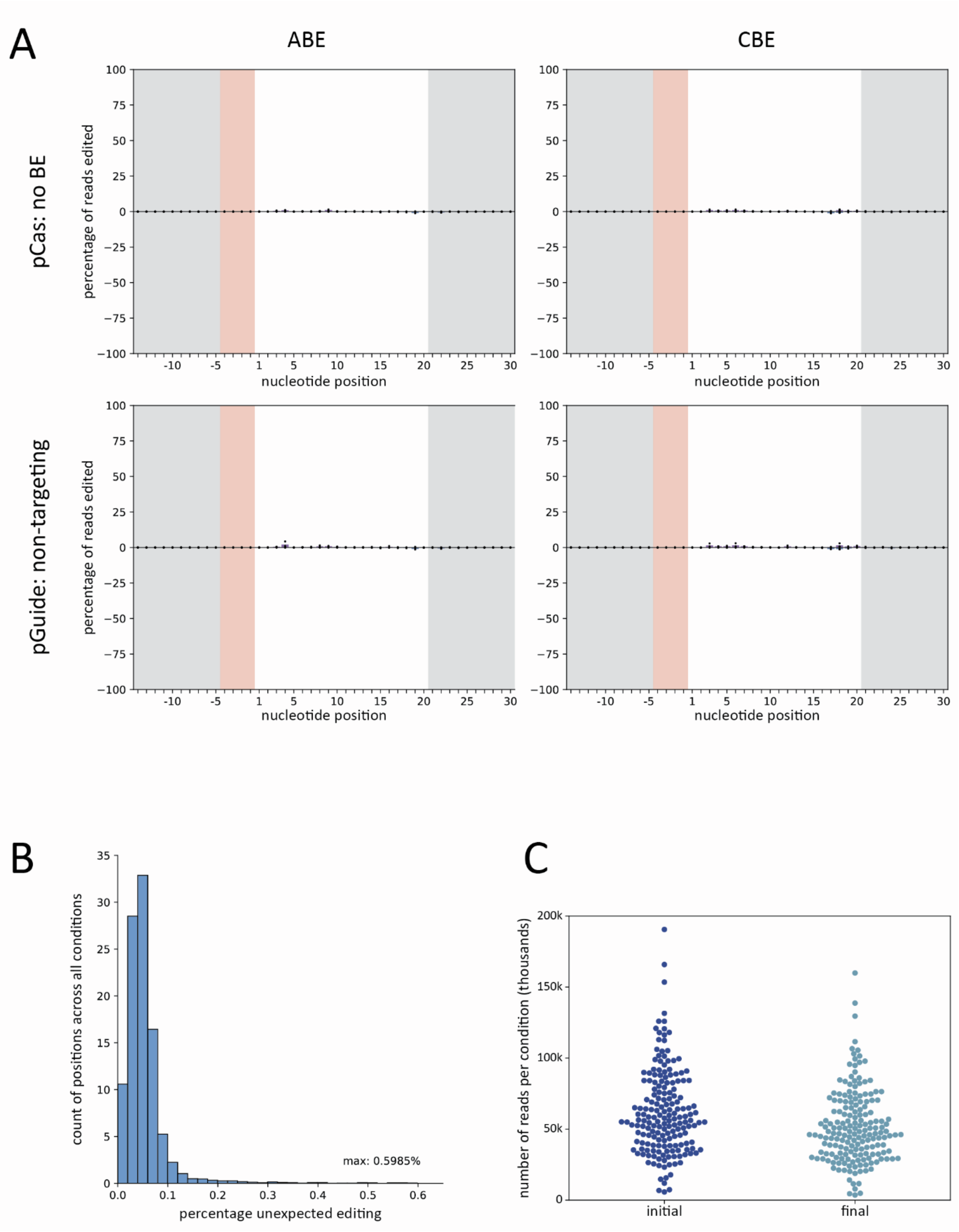

Supplementary Figure 2. Negative controls, unexpected editing, and read retention. (A) Bar-charts showing base editing activity of negative controls at tiled positions (Figure 1), combining data from all protospacers for ABE and CBE after 72h. The data from each biological replicate is shown as black dots, while bars indicate the average of the three replicates. The pink-shaded area indicates the PAM, the grey-shaded areas are upstream from the PAM and downstream from the 20nt we refer to as protospacer. Purple bars: non-target strand editing. Blue bars: target strand editing. pCas: noBE indicates that a catalytically dead AsCas12f1 was used without any deaminase domain. pGuide: non-targeting indicates that a non-targeting guide was used. **(B)** Histogram of edits other than the expected base conversions by ABE or CBE for any position across all tested conditions. The maximum 'unexpected' editing at a single position in a single condition is indicated on the bottom right

of the plot area. **(C)** Distribution of the number of sequencing reads (in thousands), with each dot representing one conditions across all three biological replicates. The initial distribution shows the numbers of reads directly after they were separated based on barcoding. The final distribution shows the numbers of reads left after cleaning and filtering.

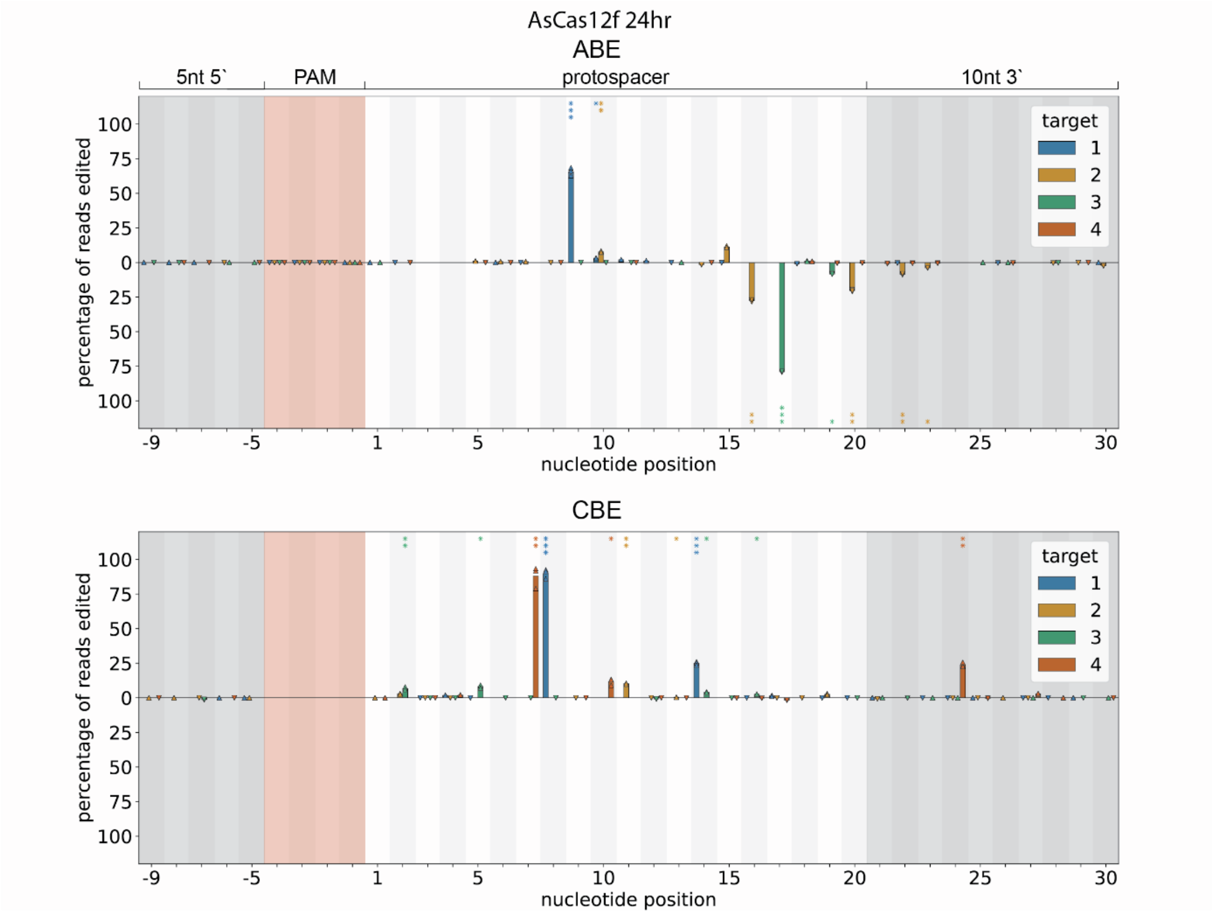

Supplementary Figure 3. ABE and CBE editing in genomic targets by AsCas12f at 24hours. Bar-charts showing base editing activity on genomic targets, combining data from four targets, 1 to 4 (supplementary table 5). The data from each biological replicate is shown as a triangle, while the bars indicate the average of the three replicates. Triangle point up or down refers to NT and T strand editing. The pink-shaded area indicates the PAM, the grey-shaded areas are upstream from the PAM and downstream of the protospacer (20nt). Above the x-axis refers to NT strand editing and below the x-axis refers to T strand editing. P-values are indicated with *:<0.05, **<0.01 and ***<0.001.

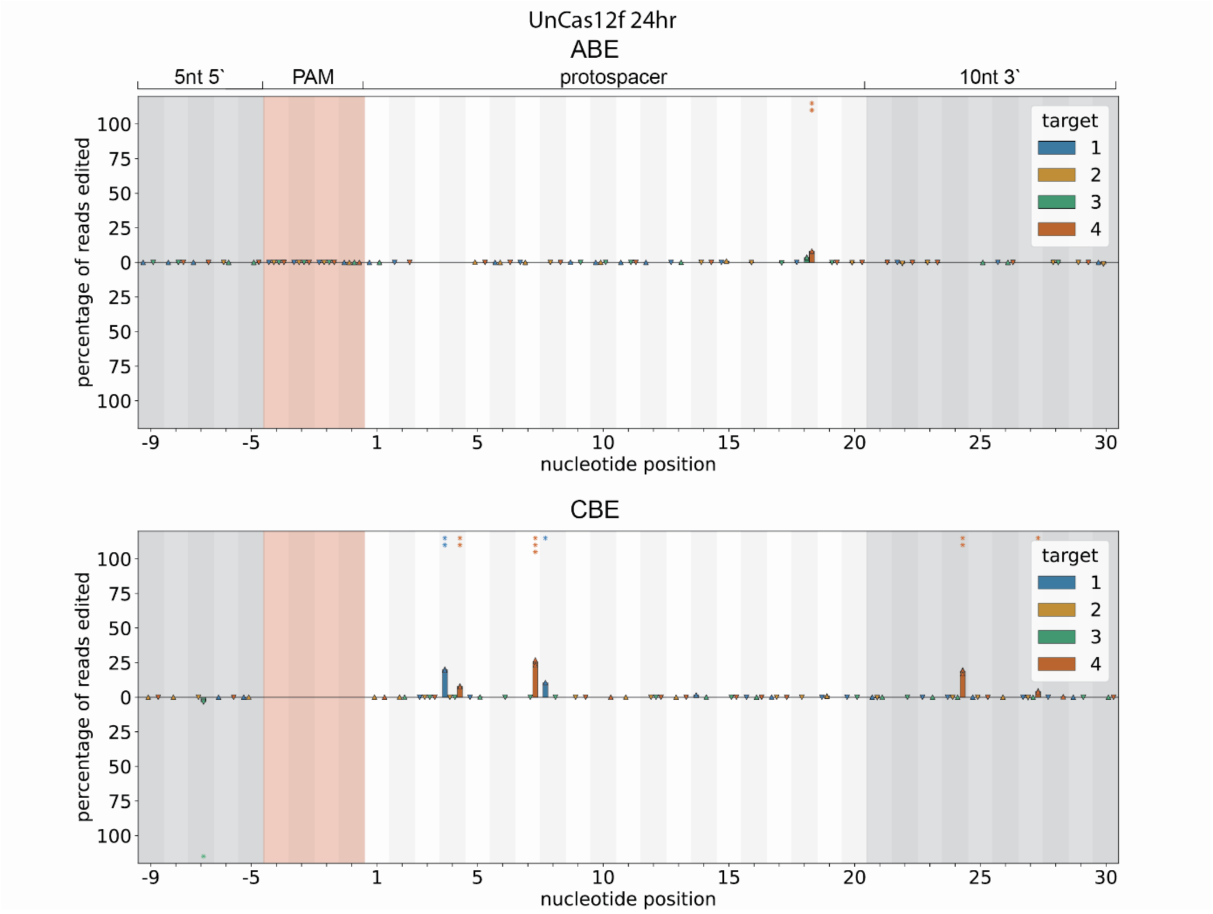

Supplementary Figure 4. ABE and CBE editing in genomic targets by Un1Cas12f at 24hours. Bar-charts showing base editing activity on genomic targets, combining data from four targets, 1 to 4 (supplementary table 5). The data from each biological replicate is shown as a triangle, while the bars indicate the average of the three replicates. Triangle point up or down refers to NT and T strand editing. The pink-shaded area indicates the PAM, the grey-shaded areas are upstream from the PAM and downstream of the protospacer (20nt). Above the x-axis refers to NT strand editing and below the x-axis refers to T strand editing. P-values are indicated with *:<0.05, **<0.01 and ***<0.001.

Supplementary Table 1. Top ten mutations identified by NGS for AsCas12f CBE editing of plasmid targets C1-6. Percentages represent the mean of three biological replicates, indicating the proportion of reads containing each mutation. Standard deviations (±) were calculated from three biological replicates.

		target	edits	percentage
CBE	24hr	C5	C10T, G17A	65.3 ±2.6%
		C1	C4T, C7T, C19T	36.5 ±0.3%
		C4	C15T, G19A	31.6 ±0.3%
		C4	C15T	18.7 ±2.4%
		C6	C14T, G18A	17.5 ±0.4%
		C5	C10T	16.8 ±2.3%
		C2	C4T, C5T	15.8 ±1.4%
		C3	C3T, C6T, C18T	15.0 ±0.3%
		C1	C4T, C7T	14.8 ±1.6%
		C6	G18A	14.2 ±0.5%
		C3	C3T, C4T, C6T, C18T	13.4 ±0.8%
	48hr	target	edits	percentage
		C5	C10T, G17A	77.6 ±0.4%
		C1	C4T, C7T, C19T	42.6 ±2.0%
		C4	C15T, G19A	27.8 ±2.6%
		C6	C14T, G18A	20.3 ±0.0%
		C2	C4T, C5T	17.8 ±0.3%
		C3	C3T, C6T, C18T	17.7 ±0.6%
		C4	C15T, G16A, G19A	15.5 ±1.6%
		C1	C4T, C7T	15.5 ±1.0%
		C3	C3T, C4T, C6T, C18T	15.2 ±0.1%
		C3	C3T, C4T, C6T, C12T, C18T	12.6 ±0.2%
		C4	None	11.5 ±7.6%
	72hr	target	edits	percentage
		C5	C10T, G17A	64.3 ±3.5%
		C4	None	40.1 ±21.7%
		C1	None	37.6 ±0.7%
		C1	C4T, C7T, C19T	27.3 ±1.9%
		C2	C4T, C5T	17.4 ±0.9%
		C3	C3T, C6T, C18T	16.7 ±1.2%
		C4	C15T, G19A	16.5 ±6.7%
		C6	C14T, G18A	15.4 ±0.8%
		C3	C3T, C4T, C6T, C18T	14.0 ±0.9%
		C5	None	13.5 ±7.0%
		C6	None	12.7 ±4.7%

Supplementary Table 2. Top ten mutations identified by NGS for AsCas12f ABE editing of plasmid targets C1-6. Percentages represent the mean of three biological replicates, indicating the proportion of reads containing each mutation. Standard deviations (±) were calculated from three biological replicates.

		target	edits	percentage
ABE	24hr	A5	None	73.6 ±2.7%
		A4	A8G, T19C, T22C	32.4 ±3.4%
		A1	A4G	24.4 ±3.2%
		A3	A9G	22.9 ±2.8%
		A4	None	22.8 ±9.2%
		A2	None	21.5 ±9.5%
		A4	A8G, T19C	21.5 ±3.4%
		A3	None	20.4 ±8.1%
		A2	T16C, T22C	19.6 ±1.5%
		A6	None	18.6 ±9.4%
		A1	None	16.5 ±9.8%
	48hr	target	edits	percentage
		A5	None	41.0 ±3.9%
		A4	A8G, T19C, T22C	37.4 ±4.4%
		A2	T16C, T22C	26.1 ±0.6%
		A1	A4G	25.9 ±1.2%
		A4	A8G, T19C	23.7 ±7.1%
		A3	A9G	23.4 ±1.0%
		A1	A4G, T18C	21.4 ±1.4%
		A5	T17C	17.9 ±1.9%
		A2	A8G	15.0 ±0.8%
		A6	A16G	14.5 ±0.5%
		A3	A3G, A9G	13.3 ±0.1%
	72hr	target	edits	percentage
		A4	A8G, T19C, T22C	38.8 ±5.2%
		A1	A4G	32.1 ±3.7%
		A5	None	27.6 ±5.4%
		A2	T16C, T22C	23.7 ±2.0%
		A1	A4G, T18C	21.5 ±1.6%
		A5	T17C	20.0 ±6.8%
		A3	A9G	17.8 ±2.9%
		A3	A9G, A12G	15.3 ±12.8%
		A4	A8G, T19C, T22C, T25C	14.8 ±2.1%
		A2	A8G	13.6 ±1.0%
		A3	A3G, A9G	12.3 ±1.8%

Supplementary Table 3. **Plasmids used in this study.** The names, and sources of the plasmids used in this study. Some plasmids have been deposited at addgene. Their accession numbers are listed in the Key Resources Table.

**Plasmid name**	**Plasmid source**
**pCas**	
Gblock AsdCas12f1 +sgRNA	this study
Gblock Un1dCas12f1 (BG35577)	this study
pCas-dCas12f1	this study
pCas-Cas12f1-CBE1	this study
pCas-Cas12f1-CBE1.2	this study
pCas-Cas12f1-ABE	this study
pCas-AsdCas12f1-LVA	this study (addgene #220993)
pCas-AsdCas12f1-CBE1_LVA	this study (addgene #220994)
pCas-AsdCas12f1-ABE_LVA	this study (addgene #220995)
pCas-Un1dCas12f1-LVA	this study
pCas-Un1dCas12f1-CBE1_LVA	this study
pCas-Un1dCas12f1-ABE_LVA	this study
**pGuide**	
pAsGuide-RFP entry	this study
pAsGuide-NT	this study
pAsGuide-C1	this study
pAsGuide-C2	this study
pAsGuide-C3	this study
pAsGuide-G1	this study
pAsGuide-G2	this study
pAsGuide-G3	this study
pAsGuide-A1	this study
pAsGuide-A2	this study
pAsGuide-A3	this study
pAsGuide-T1	this study
pAsGuide-T2	this study
pAsGuide-T3	this study
pAsGuide-rbsB set 1	this study
pAsGuide-rbsR.1	this study
pAsGuide-rbsR.2	this study
pAsGuide-arsB.2	this study
Gblock Un1Guide-RFP (BG35578)	this study
pUn1Guide-RFP entry	this study
pUn1Guide-NT	this study
pUn1Guide-rbsB set 1	this study
pUn1Guide-rbsR.1	this study
pUn1Guide-rbsR.2	this study
pUn1Guide-arsB.2	this study
**pTarget**	

pTarget-entry (divergent)	this study (addgene #220996)
pTarget-C1	this study
pTarget-C2	this study
pTarget-C3	this study
pTarget-G1	this study
pTarget-G2	this study
pTarget-G3	this study
pTarget-A1	this study
pTarget-A2	this study
pTarget-A3	this study
pTarget-T1	this study
pTarget-T2	this study
pTarget-T3	this study

Supplementary Table 4. Oligonucleotides for the assembly of pCas, pGUIDE and pTarget. The internal ID, sequence and description of each oligonucleotide used in the assembly of pCas, pGuide, and pTarget plasmids for this study.

Oligo ID	Sequence (5'-3')	Description
**Construction of the pCas12f1 plasmids**		
BG23576	GCGAGCTCAGGAGGACATC	pCas Flank L Fw
BG23577	CGCCAAAACAGCCAAGCTT	pCas Flank R Rv
BG14064	GATGTCCTCCTGAGCTCGC	pCasFlank L Rev
BG14065	AAGCTTGGCTGTTTTGGCG	pCas Flank R Fw
BG23587	AGTCTCGCTGCCGCTTTTGATGCTTTCCGCGATGATTTTATC	XTEN_Cas12f Rv
BG23590	TCTGGTGGTTCTTCTGGTGGTTCTAGCGG	XTEN Fw
BG23590	TCTGGTGGTTCTTCTGGTGGTTCTAGCGG	exXTEN Fw
BG23591	AGAAGAACCACCAGATTTGATGCTTTCCGCGATGATTTTATC	exXTEN Cas12f Rv
BG19262	AGCGGCAGCGAGACTCCCGGGACCTCAGAGTCCGCCACACCCGA	XTEN Fw
BG29726	caGAAGACtagtagctTAAAAGCTTGGCTGTTTTGGCGGATG	pCas-LVA_fw_BbsI (for ABE-LVA, CBE-LVA,noBE-LVA)
BG29727	caGAAGACagctactaaTTTGATGCTTTCCGCGATGATTTTATCGATG	pCasnoBE-LVA_rv_BbsI
BG29728	caGAAGACagctactaaGTTGATGGAGCTCTGGGCCTTC	pCas-ABE_rv_BbsI
BG29729	caGAAGACatgcTTTCCGCGATGATTTTATCGATGTTCG	pCasCBE_F1_rv_BbsI
BG29730	caGAAGACgaaaGCATCAAAAGCGGCAGCGA	pCasCBE_F2_fw_BbsI
BG29731	caGAAGACagctactaaGAGCATCTTAATCTTGTTCTCACCGTTGC	pCasCBE_F2_rv_BbsI
BG29726	caGAAGACtagtagctTAAAAGCTTGGCTGTTTTGGCGGATG	pdCasBE-LVA_fw_BbsI (ABE-LVA and CBE-LVA)
**Construction of pQUIDE plasmids**		
BG23580	CCGGCTTATCGGTCAGTTTCAC	pCRISPR backbone Fw
BG23581	GCTAGCATTATACCTAGGACTGAGCTAGC	pCRISPR bakbone Rv
BG23578	TCGGGTCGAAGACATATTCGTCGGTTCAGCGACGATAAG	sgRNA Fw
BG23579	TGACCGATAAGCCGGCTGTC	sgRNA Rv
BG23582	AGGTATAATGCTAGCATGTCTTCCCCATACGATATAAGTTGTAATTC	BbsI RFP Fw
BG23583	ATGTCTTCGACCCGAAAAGTG	Bbsi RFP Rv
BG23859	ATTCGTCGGTTCAGCGACGATAAG	sgRNA Fw
BG23860	GATGCGAAGACCAGTTCACACTCCACAAGCTAGCTC	sgRNA rv BbsI
BG23861	GTTAGAAGACAAGGCCGGCATGGTCCCAGCCTC	HDV fw BbsI
BG23880	GAACTGGTCTTCACAACGGTTTCCCTCTAGAAATAATTTTGTTTAAC	RFP BbsI Fw
BG23881	GGCCTTGTCTTCGACCCGAAAAGTGCCACTTGC	RFP BbsI Rv
BG23864	GAACTGGTCTTCGCATCTTGCCGTTAGAAGACAA	Cas12f BbsI NT Fw
BG23865	GGCCTTGTCTTCTAACGGCAAGATGCGAAGACCA	Cas12f BbsI NT Rv
BG23866	GAACCATCGTCAACGTCAGCATCT	Cas12f C motif 1 Fw
BG23867	GGCCAGATGCTGACGTTGACGATG	Cas12f C motif 1 Rv
BG23868	GAACACTCCTACAGCTACGACTTC	Cas12f C motif 2 Fw
BG23869	GGCCGAAGTCGTAGCTGTAGGAGT	Cas12f C motif 2 Rv

BG23870	GAACATCCTCAACGTCAGCATCTA	Cas12f C motif 3 Fw
BG23871	GGCCTAGATGCTGACGTTGAGGAT	Cas12f C motif 3 Rv
BG23874	GAACACCACTATCAGCACGAGTAT	Cas12f A motif 1 Fw
BG23875	GGCCATACTCGTGCTGATAGTGGT	Cas12f A motif 1 Rv
BG23876	GAACCACAATTACGACTATTAGGA	Cas12f A motif 2 Fw
BG23877	GGCCTCCTAATAGTCGTAATTGTG	Cas12f A motif 2 Rv
BG23878	GAACCCAATATTAGCATGATCAGC	Cas12f A motif 3 Fw
BG23879	GGCCGCTGATCATGCTAATATTGG	Cas12f A motif 3 Rv
BG25857	GAACGTAGCAGTTGCAGTCGTAGA	cas12f1 G1 tile Fw
BG25858	GGCCTCTACGACTGCAACTGCTAC	cas12f1 G1 tile Rv
BG25859	GAACTGAGGATGTCGATGCTGAAG	cas12f1 G2 tile Fw
BG25860	GGCCCTTCAGCATCGACATCCTCA	cas12f1 G2 tile Rv
BG25861	GAACTAGGAGTTGCAGTCGTAGAT	cas12f1 G3 tile Fw
BG25862	GGCCATCTACGACTGCAACTCCTA	cas12f1 G3 tile Rv
BG25863	GAACTGGTGATAGTCGTGCTCATA	cas12f1 T1 tile Fw
BG25864	GGCCTATGAGCACGACTATCACCA	cas12f1 T1 tile Rv
BG25865	GAACGTGTTAATGCTGATAATCCT	cas12f1 T2 tile Fw
BG25866	GGCCAGGATTATCAGCATTAACAC	cas12f1 T2 tile Rv
BG25867	GAACGGTTATAATCGTACTAGTCG	cas12f1 T3 tile Fw
BG25868	GGCCCGACTAGTACGATTATAACC	cas12f1 T3 tile Rv
**Construction of pTarget Plasmids**		
BG28941	CGTCTTCAGCtttaCATCGTCAACGTCAGCATCTGCAGCTACGAgtac	C1_displaced_FW
BG28942	TCGTAGCTGCAGATGCTGACGTTGACGATGtaaaGCTGAAGACGacgt	C1_displaced_RV
BG28943	GCTACTGCGAtttaACTCCTACAGCTACGACTTCGTCGACAGCAgtac	C2_displaced_FW
BG28944	TGCTGTCGACGAAGTCGTAGCTGTAGGAGTtaaaTCGCAGTAGCacgt	C2_displaced_RV
BG28945	GGCATCGACAtttaATCCTCAACGTCAGCATCTACTACATCGGCgtac	C3_displaced_FW
BG28946	GCCGATGTAGTAGATGCTGACGTTGAGGATtaaaTGTCGATGCCacgt	C3_displaced_RV
BG28947	GCTGTTGACGtttaGTAGCAGTTGCAGTCGTAGACGACGTAGCAgtac	G1_target_FW
BG28948	TGCTACGTCGTCTACGACTGCAACTGCTACtaaaCGTCAACAGCacgt	G1_target_RV
BG28949	CGTAGTCGCAtttaTGAGGATGTCGATGCTGAAGCTGCAGACGAgtac	G2_target_FW
BG28950	TCGTCTGCAGCTTCAGCATCGACATCCTCAtaaaTGCGACTACGacgt	G2_target_RV
BG28951	CCGATGCAGAtttaTAGGAGTTGCAGTCGTAGATGTAGATGCCGgtac	G3_target_FW
BG28952	CGGCATCTACATCTACGACTGCAACTCCTAtaaaTCTGCATCGGacgt	G3_target_RV
BG28953	AGTATTACGAtttaACCACTATCAGCACGAGTATGACGATCAGCgtac	A1_displaced_FW
BG28954	GCTGATCGTCATACTCGTGCTGATAGTGGTtaaaTCGTAATACTacgt	A1_displaced_RV
BG28955	GATCATGAGCtttaCACAATTACGACTATTAGGAGTAGCACGACgtac	A2_displaced_FW
BG28956	GTCGTGCTACTCCTAATAGTCGTAATTGTGtaaaGCTCATGATCacgt	A2_displaced_RV
BG28957	GGACTAGCACtttaCCAATATTAGCATGATCAGCATCACTAGGAgtac	A3_displaced_FW
BG28958	TCCTAGTGATGCTGATCATGCTAATATTGGtaaaGTGCTAGTCCacgt	A3_displaced_RV
BG28959	TCGTGGTACTtttaTGGTGATAGTCGTGCTCATACTACTGATCAgtac	T1_target_FW
BG28960	TGATCAGTAGTATGAGCACGACTATCACCAtaaaAGTACCACGAacgt	T1_target_RV
BG28961	CTGATGCTCAtttaGTGTTAATGCTGATAATCCTCGTCATACTAgtac	T2_target_FW
BG28962	TAGTATGACGAGGATTATCAGCATTAACACtaaaTGAGCATCAGacgt	T2_target_RV
BG28963	CCTAGTCATAtttaGGTTATAATCGTACTAGTCGTGATAGTCCTgtac	T3_target_FW
BG28964	AGGACTATCACGACTAGTACGATTATAACCtaaaTATGACTAGGacgt	T3_target_RV

Supplementary Table 5. **Sequences and loci of *E. coli* genomic targets.** The genomic targets as used in the assessment of chromosomal base editing.

**No**	**Loci**	**Spacer Sequence (5'->3')**	**PAM**
1	rbsB	atgcgatcaaaatctgctgg	ttta
2	rbsR	ccggaaatgacgctatggct	ttta
3	rbsR	acggcgggtttgacgctatg	ttta
4	arsB	ctgcttctgctggtgggatt	ttta
